# Ataxia-telangiectasia: immunologic profile and clinical outcome

**DOI:** 10.1186/1939-4551-8-S1-A203

**Published:** 2015-04-08

**Authors:** Clarissa De Lima Honório Dumas, Fabiola Scancetti Tavares, Hermínio De Paula Ramos Netto, Vanessa Silva Pereira Campos, Cláudia França Cavalcante Valente, Luciana Lilian Dias Pereira

**Affiliations:** 1 Asbai, Brazil; 2 Hospital De Base Do Distrito Federal, Brazil

## Background

Evaluate the immunologic profile and clinical outcome of Ataxia-Telangiectasia (AT) patients followed on a immunology service of a health unity in Brasilia.

## Methods

This is a retrospective study and the informations were retrieved from the patients medical records.

## Results

In our service, a hundred and thirty patients with the diagnosis of a primary immunodeficiency are followed-up and from this sample we have four patients with AT. Acessing clinical history and the laboratory findings becomes clear that a large variability occurs concerning their immune system.

All subjects presented recurrent infections, especially sinopulmonary . In laboratory evaluation it was shown immunoglobulin A (IgA) deficiency, lymphopenia due to low lymphocyte T count and selective antibody deficiency with normal immunoglobulins levels. Some of these patients also require human immunoglobulin replacement and two of them evolved with lymphoid malignancy (Hodgkin and non-Hodgkin lymphoma).

## Conclusions

This study demonstrates that clinical aspects and level of immunodeficiency has a large variation and that both cellular and humoral immunity might be affected. This presentation is the same one found in databases worldwide.

A multidisciplinary approach allows adequate control of infectious episodes and related comorbidities with a positive impact on their quality of life.

